# circRNA Expression Pattern and circRNA–miRNA–mRNA Network in HCs, HSCs, and KCs of Murine Liver After *Echinococcus multilocularis* Infection

**DOI:** 10.3389/fvets.2022.825307

**Published:** 2022-03-24

**Authors:** Tingli Liu, Liqun Wang, Hong Li, Yanping Li, Guoliang Chen, Guiting Pu, Xiaola Guo, Yadong Zheng, Xue Bai, Xuenong Luo

**Affiliations:** ^1^State Key Laboratory of Veterinary Etiological Biology, Key Laboratory of Veterinary Parasitology of Gansu Province, Lanzhou Veterinary Research Institute, Chinese Academy of Agricultural Sciences (CAAS), Lanzhou, China; ^2^Key Laboratory of Applied Technology on Green-Eco Healthy Animal Husbandry of Zhejiang Province, Zhejiang Provincial Engineering Laboratory for Animal Health Inspection and Internet Technology, College of Animal Science and Technology and College of Veterinary Medicine, Zhejiang A&F University, Hangzhou, China; ^3^Key Laboratory for Zoonoses Research, Ministry of Education, College of Veterinary Medicine, OIE Collaborating Center on Foodborne Parasites in Asian-Pacific Region, Institute of Zoonoses, Jilin University, Changchun, China

**Keywords:** *Echinococcus multilocularis*, HCs, HSCs, KCs, circRNA expression, circRNA-miRNA-mRNA network

## Abstract

Caused by *Echinococcus multilocularis* (*E. multilocularis*), alveolar echinococcosis is reported every year around the world and severely threatens the safety of human beings and animals. However, the molecular interaction relationships between host and *E. multilocularis* still remains unclear. With multiple functions, circRNA plays a crucial role in regulating the development of a parasitic disease. With that in mind, the main purpose of this study was to reveal the circRNA expression profiles and circRNA–miRNA–mRNA network relationships in hepatocytes (HCs), hepatic stellate cells (HSCs), and Kupffer cells (KCs) of murine liver after *E. multilocularis* infection. After sequencing, 6,290 circRNAs were identified from 12 hepatic cell samples. Based on the subsequent analysis, 426 and 372 circRNAs were significantly different in HC expression at 2 and 3 months after *E. multilocularis* infection, and similar results were also demonstrated in HSCs (426 and 372 circRNAs) and KCs (429 and 331 circRNAs), respectively. Eight candidate circRNAs were randomly selected to identify the accuracy of the sequencing results by using qRT-PCR. Additionally, three circRNAs–miRNA–mRNA networks in HCs, HSCs, and KCs were constructed. Taken together, our study provided a systematic presentation of circRNAs in murine liver cells after *E. multilocularis* infection, and these networks are essential for research in circRNAs associated with *E. multilocularis* infection.

## Introduction

With high morbidity and mortality, alveolar echinococcosis (AE) primarily affects the liver and lung in humans and other mammals. As the pathogen of AE, *Echinococcus multilocularis* (*E. multilocularis*) has been considered as the top-ranking food-borne parasite in Europe (1). At present, except for surgical removal or benzimidazole–carbamate treatment, no other efficient therapeutics were discovered. However, surgical removal is usually accompanied by a high recurrence rate, and there is limited potential for the derivative to recover fully (2). Thus, further understanding on the mechanism of interaction between the host and *E. multilocularis* will be more propitious to reveal the pathogenic mechanism and can accelerate the development of new therapeutics for the disease.

As a kind of ncRNA back-spliced by host genes, circular RNA (circRNA) is stable due to its lack of naked 5′ and 3′ ends. Generally, three types of circRNAs were identified, including exon-circRNA, exon-intron-circRNA, and intron-circRNA. With the development of next-generation sequencing and bioinformatics analysis, circRNAs have received more and more attention due to its dysregulated expression being associated with diseases (3–7). Participating in transcriptional and post-translational activities, circRNAs are employed as RNA binding protein sponges, miRNA sponges, and protein scaffolds in various biological processes (7–9). Besides this, translational function has been proved in circRNAs, and these findings enrich the understanding of circRNA (10). Among them, competitive endogenous RNA (ceRNA) regulation is an important pattern in the circRNA regulation mechanism. As the name implies, in the ceRNA mechanism, circRNAs competes with common targets for miRNAs to regulate the expression of targets, which has been verified by many researchers—for instance, circRNA-100876 could promote the metastasis and proliferation of gastric cancer by regulating the MIEN1 expression, and the regulated expression of MIEN1 was achieved by sponging miR-136 (11). Serving as a sponge for miR-92b-3p, circPSD3 could mediate the expression of Smad7, leading to alleviated hepatic fibrogenesis (12). Based on this, circRNAs play an important role in a variety of pathological processes, while in the interaction between the host and *E. multilocularis*, the roles of circRNAs are still unevaluated.

As the predilection tissue of *E. multilocularis*, the liver will undergo a series of pathological changes after an infection, such as hepatic granulomatous inflammation response (13), fibrosis, and even liver failure (14, 15). Such pathological changes are related to various cells in the liver. Of them, the hepatocytes are the basic unit of the liver, mainly responsible for synthesis, metabolism, and secretion function. Under a normal condition, hepatic stellate cells (HSCs) participate in the regulation of extracellular matrix homeostasis, vasoregulation, and retinoid storage. Once the injuries occurred, the HSCs can generate collagen and destroy the extracellular matrix to regulate fibrosis and then maintain homeostasis. As the macrophages reside in the liver, Kupffer cells could correspond to the immune system by antigen procession and presentation. Taken together, the different cells in the liver play various roles after *E. multilocularis* infections, so what role circRNAs play in HCs, HSCs, and KCs is worth studying.

Based on this, we successfully isolated and identified HCs, HSCs, and KCs from infected and uninfected murine at 2 and 3 months post-infection (mpi). Combined with circRNA sequencing, the expression patterns and possible functions of differentially expressed circRNAs in different cells originated from murine liver after *E. multilocularis* infections were revealed, circRNA–miRNA–mRNA networks were constructed, and these findings would shed light on the subsequent studies on AE.

## Materials and Methods

### Construction of *E. Multilocularis*-Infected Murine Model

Thirty specific pathogen-free BALB/c murine (6 weeks old, male) were randomly divided into two groups, at 15 individuals per group. In total, 600 protoscolices, which were separated from the abdominal cavity of Mongolian gerbil as previously reported (16), were administrated per murine in one group (named E) by intraperitoneal injection, while the same volume of phosphate-buffered saline was given to another group (named C), respectively. The same feeding environment was given to the two groups.

### Isolation and Identification of HCs, HSCs, and KCs of Murine

In order to get a sufficient number of HSCs and KCs in each group, three kinds of main cells from the murine liver of six murines after 2 and 3 mpi were collected using collagenase digestion and density gradient centrifugation. In short, 0.04 and 0.08% collagenase IV (GIBICO, USA) were used to digest the liver to obtain a cell mixture. Then, the HCs in pellet were isolated by centrifugation at 50 × *g*. The supernatant containing HSCs and KCs was further separated using 20 and 11.5% OptiPrep solution by centrifugation at 1,400 × *g*. To identify the purity of three kinds of cells, qRT-PCR was applied to detect the expression level of the surface markers of different cells.

### Preparation of Sequencing Library and Identification of circRNA

The total RNA of different cells was isolated using TRIzol reagent (Invitrogen, Carlsbad, CA, USA). After detecting the integrity of RNA, library preparation and sequencing were performed by BGI Biotechnology Corporation (Wuhan, China). The purified RNA was treated with Ribo-Zero^TM^ Magnetic Kit (Epicenter Biotechnologies, Madison, WI, USA) to eliminate ribosomal RNA, followed by cDNA synthesis, 3′ adenylate, adapters ligate, cDNA enrichment by PCR amplification, and purification of PCR products. Then, the final library was checked in terms of the size distribution and quantitated for sequencing. Raw reads were generated subsequently, which were deposited in NCBI Sequence Read Archive (SRA) under accession number PRJNA732233. To obtain high-quality data (clean reads), the reads with adaptor, unknown base content of more than 5%, and low-quality reads were removed. Subsequently, the clean reads were mapped into the *Mus musculus* genome (GCF_000001635.26_GRCm38.p6) to gain circRNAs. Fragments per kilobase of transcript per million mapped reads (FPKM) algorithm was used to quantitatively normalized the expression of circRNAs. Differentially expressed circRNAs (DECs) with statistical significance between the E and C groups were identified by analyzing the log2 |E/C| and FDR (E/C), in accordance with the log2|E/C| ≥1 and FDR (E/C) ≤0.001 standard.

### Interaction Between circRNA, miRNA, and mRNA

It is crucial to look for the target genes of DECs to understand their function. By analyzing the target site of DECs–miRNAs and miRNAs–mRNAs, the potential relationship between them was identified. We used TargetScan database to predict the potential target genes of targeting miRNAs. Of these, some candidate DECs were selected to construct the network of circRNA–miRNA–mRNA by using Cytoscape 3.6.1 software, which provides an intuitive way to understand the interaction.

### Utilizing qRT-PCR

Real-time quantitative reverse transcription polymerase chain reaction (qRT-PCR) was performed to verify the accuracy of sequencing. Eight DECs were randomly selected for qRT-PCR, with the primers listed in Table 1. Similarly, the total RNA of another 12 murine cell samples from 2 and 3 mpi was extracted using TRIzol reagent according to the manufacturer's instructions. After cDNA synthesis, the qRT-PCR was carried out by ABI 7500 system (Thermo Fisher Scientific, Waltham, MA, USA), with the reaction condition as follows: 95°C for 10 min and 40 cycles of 95°C for 15 s and 60°C for 1 min. The 2^−ΔΔCT^ method was utilized to obtain the relative expression level of DECs, with glyceraldehyde-3-phosphate dehydrogenase (GAPDH) as the internal reference gene.

**Table 1:** The primers of circRNAs used for qRT-PCR validation.

**circRNA-id**	**Forward primer**	**Reverse primer**
mmu-circ-0007949	TAGGCAGGTGGGAGATGAT	CGGTCTTCTGGCTCAAAT
mmu-circ-0013550	TACCTGGACTGTGTCATCAAG	TCTCACGGATAGCCTTCTCT
mmu-circ-0003295	TTTGGGAAGGGAACAGTG	CAAGTGATGGGTCCAGTG
mmu-circ-0010217	TCCCTTATCCAGTGGCGA	TGGTCTCCTCTCTCATTCAG
mmu-circ-0000539	TGTCATCGCCCTCACCTT	GAGCCAATGATGTTCCACAA
mmu-circ-0003636	AAGGCGACGATGCGAACT	TAGGTGACAGGCTATGGGC
mmu-circ-0012265	ATGGTGAGGGTGGTTACC	TGTAGGCGACAGGCTTAG
mmu-circ-0006979	GATTGAGAAAAGTAGGTTTTGTG	GTTTTAATCTACAAGGCCGACCTT

## Results

### Identification of the Purity of HCs, HSCs, and KCs

**Figure 1:**
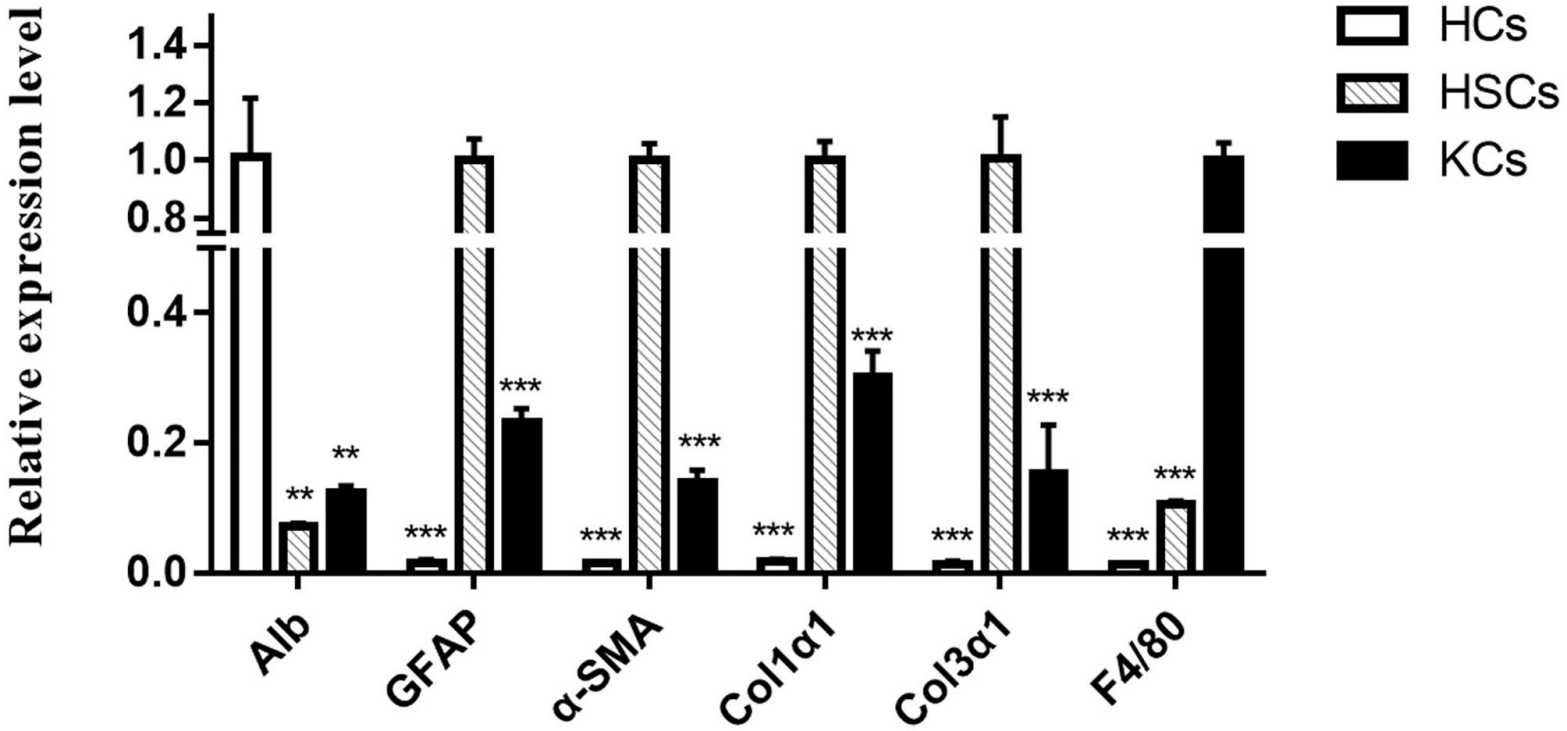
The relative expression levels of different cell markers in hepatocytes, hepatic stellate cells, and Kupffer cells were determined by qRT-PCR. The number of biological replicates for each experiment was 3, and the relative expression levels were normalized to the expression levels of GAPDH. Data are presented as means with SD. *P*-values were analyzed by Student's *t*-test. ^***^P < 0.001 and ^**^P < 0.01.

The high purity of cells is the basis of further research. In this study, we detected the cell purity of three kinds of cells using qRT-PCR. As expected, compared with the other two kinds of cells, each kind of cells has a relatively high purity (Figure 1). Among them, cytokeratin 18 (CK18), which is the cell marker of HCs, is most enriched in HCs rather than HSCs and KCs. Similarly, a-smooth muscle actin (a-SMA), collagen type 1 Alpha 1 (col1a1), collagen type 3 Alpha 1 (col3a1), and glial fibrillary acidic protein in HSCs, and mouse EGF-like module-containing mucin-like hormone receptor-like (EMR1 or F4/80) are enriched in KCs. According to this, 12 samples were separated from murine infected with *E. multilocularis* at 2 and 3 mpi to construct a library for sequencing.

### Identification of circRNA in HCs, HSCs, and KCs

The circRNA expression in HCs, HSCs, and KCs after *E. multilocularis* infection was investigated using high-throughput sequencing of 12 samples. After removal of the low-quality reads, 95.9 GB clean reads were obtained for submission to the SRA database, with access number PRJNA732233.

**Figure 2:**
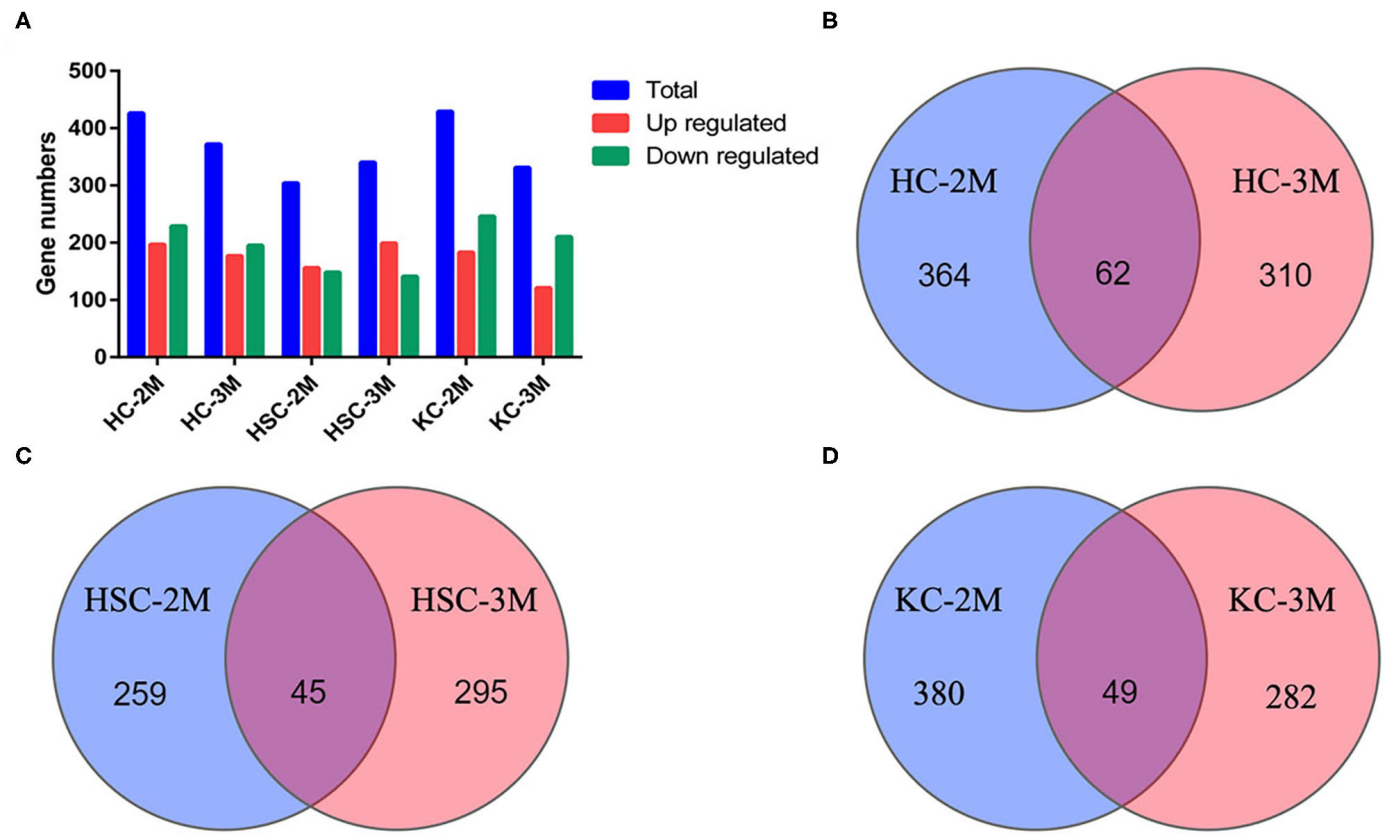
Analysis of differentially expressed circRNAs (DECs) between control and infection groups in three kinds of cells. **(A)** Number of DECs in six groups. Blue bars represent total DECs, red bars represent upregulated circRNAs, and green bars represent downregulated circRNAs. **(B)** Venn diagrams of DECs in HC-2M and HC-3M. **(C)** Venn diagrams of DECs in HSC-2M and HSC-3M. **(D)** Venn diagrams of DECs in KC-2M and KC-3M.

In total, 6,290 circRNAs were identified by high-throughput sequencing in all samples. Among these circRNAs, 4,082 circRNAs were detected in HCs, 5,332 in HSCs, and 4,671 in KCs, respectively. Based on the FPKM, the expression abundance of circRNAs of different samples was measured (Supplementary Table S1). The expression of circRNA analysis revealed that 426 circRNAs were differentially expressed in HCs at 2-mpi [log2 |(HC-2M-E/HC-2M-C)| ≥1 and FDR (HC-2M-E/HC-2M-C) ≤0.001], including 197 and 229 that were up- and downregulated, respectively, while in HCs at 3 mpi, 372 circRNAs were differentially expressed, including 177 and 195 that were up- and downregulated, respectively (Figure 2A). Among these, 62 circRNAs were differentially expressed in both 2-mpi and 3-mpi groups (Figure 2B). In HSCs, 304 circRNAs (156 up- and 148 down-regulated) were differentially expressed at 2-mpi and 340 (199 up- and 141 down-regulated) at 3-mpi (Figure 2A), with 45 circRNAs were differentially expressed in both 2-mpi and 3-mpi groups (Figure 2C). And in KCs, 429 circRNAs (183 up- and 246 down-regulated) were differentially expressed at 2-mpi and 331 (121 up- and 210 down-regulated) at 3-mpi (Figure 2A), with 49 circRNAs were differentially expressed in both the 2- and 3-mpi groups (Figure 2D). The top 10 dysregulated circRNAs of HCs, HSCs, and KCs based on log2(E/C) are summarized in Tables 2–4, respectively.

**Table 2:** Top 10 dysregulated circRNAs in hepatocytes of murine liver after *E. multilocularis* infection.

**Group**	**circRNA ID**	**log2 (E/C)**	**FDR (E/C)**	**Status**
2M-HC-E/	mmu_circ_0007969	−11.61300668	2.19E-30	Down
2M-HC-C	mmu_circ_0006755	−10.2625647	2.89E-11	Down
	mmu_circ_0016100	−10.11048331	4.79E-10	Down
	mmu_circ_0011522	−10.02804418	1.80E-09	Down
	mmu_circ_0013261	−10.02804418	1.84E-09	Down
	mmu_circ_0008494	−10.02804418	1.88E-09	Down
	mmu_circ_0011428	9.932805294	1.29E-08	Up
	mmu_circ_0008007	9.845333228	4.40E-08	Up
	mmu_circ_0014656	9.845333228	4.32E-08	Up
	mmu_circ_0005837	9.799605422	8.23E-08	Up
3M-HC-E/	mmu_circ_0001047	10.52864945	4.23E-12	Up
3M-HC-C	mmu_circ_0012970	10.19155278	2.16E-09	Up
	mmu_circ_0015623	10.15317199	4.00E-09	Up
	mmu_circ_0014690	−9.917819589	1.60E-07	Down
	mmu_circ_0002411	−9.917819589	1.66E-07	Down
	mmu_circ_0009306	9.897845456	1.73E-07	Up
	mmu_circ_0010219	−9.865269307	2.75E-07	Down
	mmu_circ_0006031	−9.865269307	2.83E-07	Down
	mmu_circ_0001764	−9.865269307	2.67E-07	Down
	mmu_circ_0003418	9.850499414	2.70E-07	Up

**Table 3:** Top 10 dysregulated circRNAs in hepatic stellate cells of murine liver after *E. multilocularis* infection.

**Group**	**circRNA ID**	**log2 (E/C)**	**FDR (E/C)**	**Status**
2M-HSC-E/	mmu_circ_0008014	−9.917073663	5.81E-10	Down
2M-HSC-C	mmu_circ_0012771	9.797661526	7.86E-09	Up
	mmu_circ_0004418	9.715275646	2.66E-08	Up
	mmu_circ_0005621	−9.614525815	6.56E-08	Down
	mmu_circ_0000992	9.534691959	3.14E-07	Up
	mmu_circ_0002861	−9.514911265	2.44E-07	Down
	mmu_circ_0006574	−9.514911265	2.60E-07	Down
	mmu_circ_0000142	−9.514911265	2.52E-07	Down
	mmu_circ_0007310	−9.462502273	4.48E-07	Down
	mmu_circ_0008203	−9.462502273	4.36E-07	Down
3M-HSC-E/	mmu_circ_0007969	11.11458826	2.89E-21	Up
3M-HSC-C	mmu_circ_0004620	10.35071823	6.21E-12	Up
	mmu_circ_0013297	10.11465333	6.51E-10	Up
	mmu_circ_0011991	9.944712061	1.16E-08	Up
	mmu_circ_0002846	9.832257114	6.28E-08	Up
	mmu_circ_0012416	9.710289863	3.31E-07	Up
	mmu_circ_0006025	9.622783966	9.40E-07	Up
	mmu_circ_0010887	9.529625764	2.49E-06	Up
	mmu_circ_0005532	9.529625764	2.57E-06	Up
	mmu_circ_0002430	9.480790201	3.71E-06	Up

**Table 4:** Top 10 dysregulated circRNAs in Kupffer cells of murine liver after *E. multilocularis* infection.

**Group**	**circRNA ID**	**log2 (E/C)**	**FDR (E/C)**	**Status**
2M-KC-E/	mmu_circ_0000645	−10.7940909	1.92E-16	Down
2M-KC-C	mmu_circ_0003772	−10.30378075	2.14E-11	Down
	mmu_circ_0009326	−10.23338001	8.46E-11	Down
	mmu_circ_0009583	−10.19684798	1.59E-10	Down
	mmu_circ_0006011	−10.12088584	6.08E-10	Down
	mmu_circ_0013598	−10.08135028	1.15E-09	Down
	mmu_circ_0010124	−10.04070069	2.19E-09	Down
	mmu_circ_0005380	−9.998872454	4.32E-09	Down
	mmu_circ_0005863	9.959277506	1.21E-08	Up
	mmu_circ_0010097	−9.955795187	8.52E-09	Down
3M-KC-E/	mmu_circ_0007969	11.58524425	1.13E-26	Up
3M-KC-C	mmu_circ_0000185	−10.12373344	1.54E-09	Down
	mmu_circ_0008942	−10.12373344	1.58E-09	Down
	mmu_circ_0016405	−10.03631101	6.08E-09	Down
	mmu_circ_0004276	−9.990529475	1.24E-08	Down
	mmu_circ_0015434	−9.943247396	2.28E-08	Down
	mmu_circ_0014830	−9.943247396	2.23E-08	Down
	mmu_circ_0015860	−9.791162889	1.83E-07	Down
	mmu_circ_0011992	9.748696028	6.78E-07	Up
	mmu_circ_0003359	9.748696028	6.88E-07	Up

### Delineation of CircRNA–MiRNA–MRNA Associations

**Figure 3:**
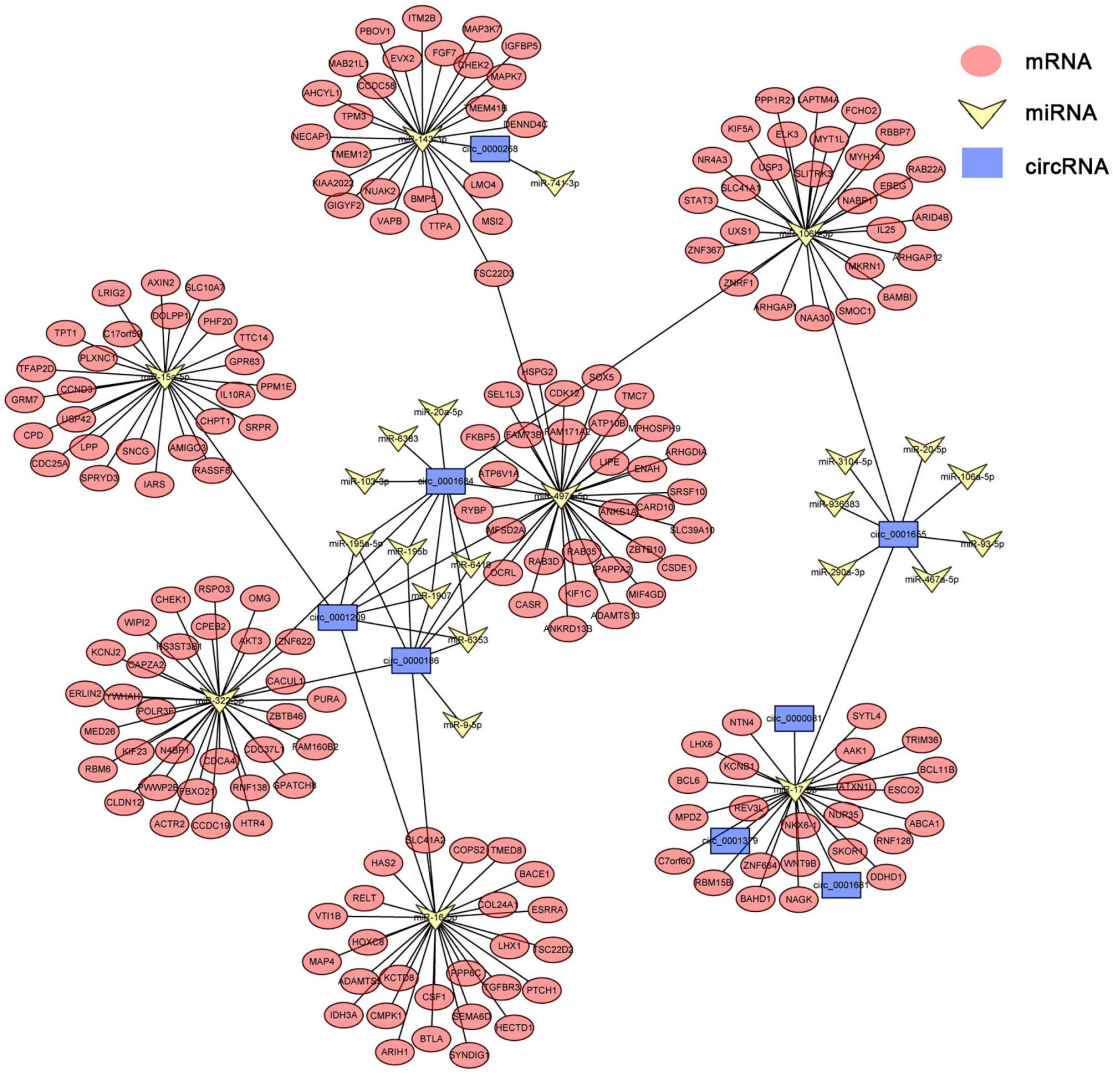
circRNA–miRNA–mRNA network diagram of hepatocytes. Blue, yellow, and red nodes correspond to circRNAs, miRNAs, and mRNAs, respectively. The black lines correspond to the interactions between RNAs.

**Figure 4:**
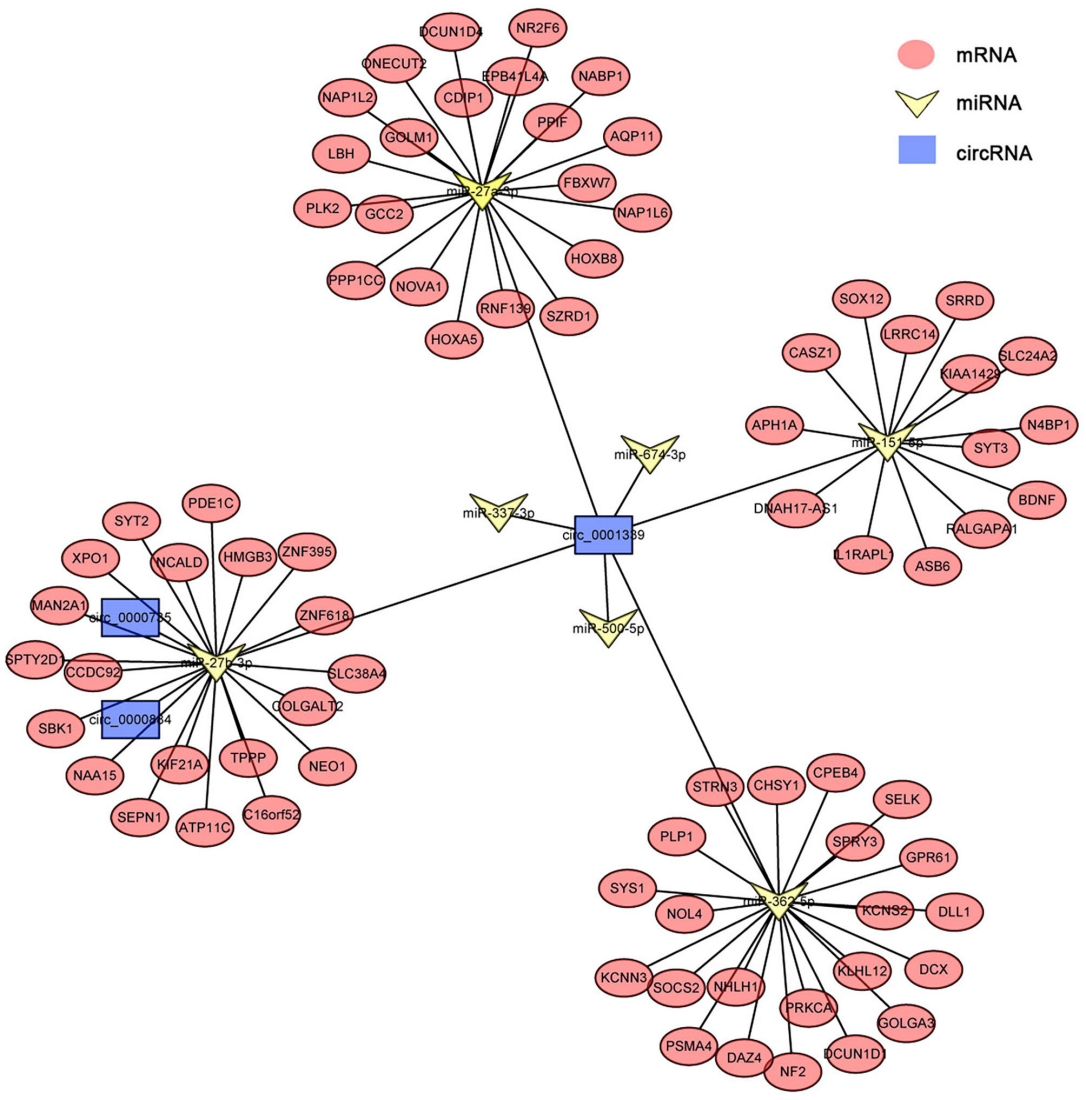
circRNA–miRNA–mRNA network diagram of hepatic stellate cells. Blue, yellow, and red nodes correspond to circRNAs, miRNAs and mRNAs, respectively. The black lines correspond to the interactions between RNAs.

**Figure 5:**
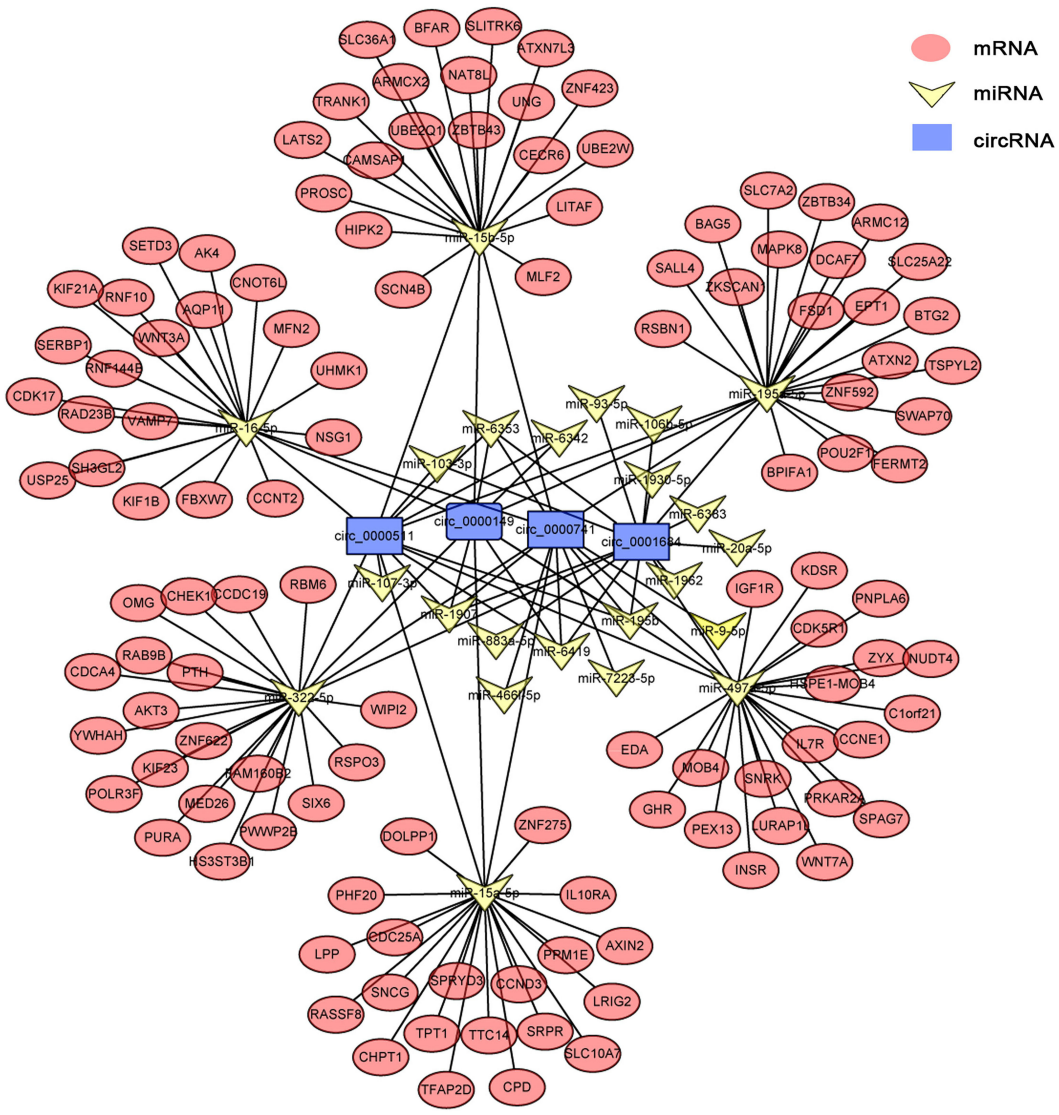
circRNA–miRNA–mRNA network diagram of Kupffer cells. Blue, yellow, and red nodes correspond to circRNAs, miRNAs, and mRNAs, respectively. The black lines correspond to the interactions between RNAs.

circRNAs, as the sponges of miRNAs, play the key role in different biological processes by inhibiting miRNA expression (17). To fully understand the potential mechanism of circRNAs, we predicted the target miRNAs interacting with some DECs based on miRanda and starBase databases. The circRNA–miRNA network for the eight DECs in HCs was constructed by using Cytoscape 3.6.1 software. The results showed that DECs could regulate many target miRNAs and downstream mRNAs (Figure 3, Supplementary Table S2)—for instance, circ-0001684 could compete with some mRNAs for miR-497-5p and miR-322-5p. In turn, miRNAs are regulated by multiple DECs, such as the association of miR-17-5p-circ-0000081, miR-17-5p-circ-0001379, and miR-17-5p-circ-0001681. It is notable that the significantly downregulated miRNA in murine liver after *E. multilocularis* infection, miR-143-3p (18), is listed as the node between circ-0000268 and MAPK7/ITM2B/NECAP1/BMP5/TSC22D3/NUAK2, which provides the possible action means of circ-0000268 and reasons of miR-143-3p downregulation expression. In addition, a similar network of circRNA–miRNA–mRNA in HSCs and KCs was constructed (Figures 4, 5, Supplementary Tables S3, S4). In the networks of HSCs, there are two significantly downregulated miRNAs in murine liver after *E. multilocularis* infection (miR-27a-3p and miR-151-5p) (18). We summarized several basic interactions involving these 2 miRNAs. circ-0001339–miR-27a-3p–FBXW7/NOVA1/GCC2/NABP1/PLK2 is an interaction based on downregulated miR-27a-3p, and circ-0001339–miR-151-5p–APH1A/IL1RAPL1/ASB6/SRRD/SYT3 is the other interaction involving ceRNAs. Based on these, circRNAs play the important regulation role by sponging miRNAs, which provide the potential regulatory mechanism in HSCs and KCs after *E. multilocularis* infection.

### Verification of the Accuracy of circRNA-Seq Data

**Figure 6:**
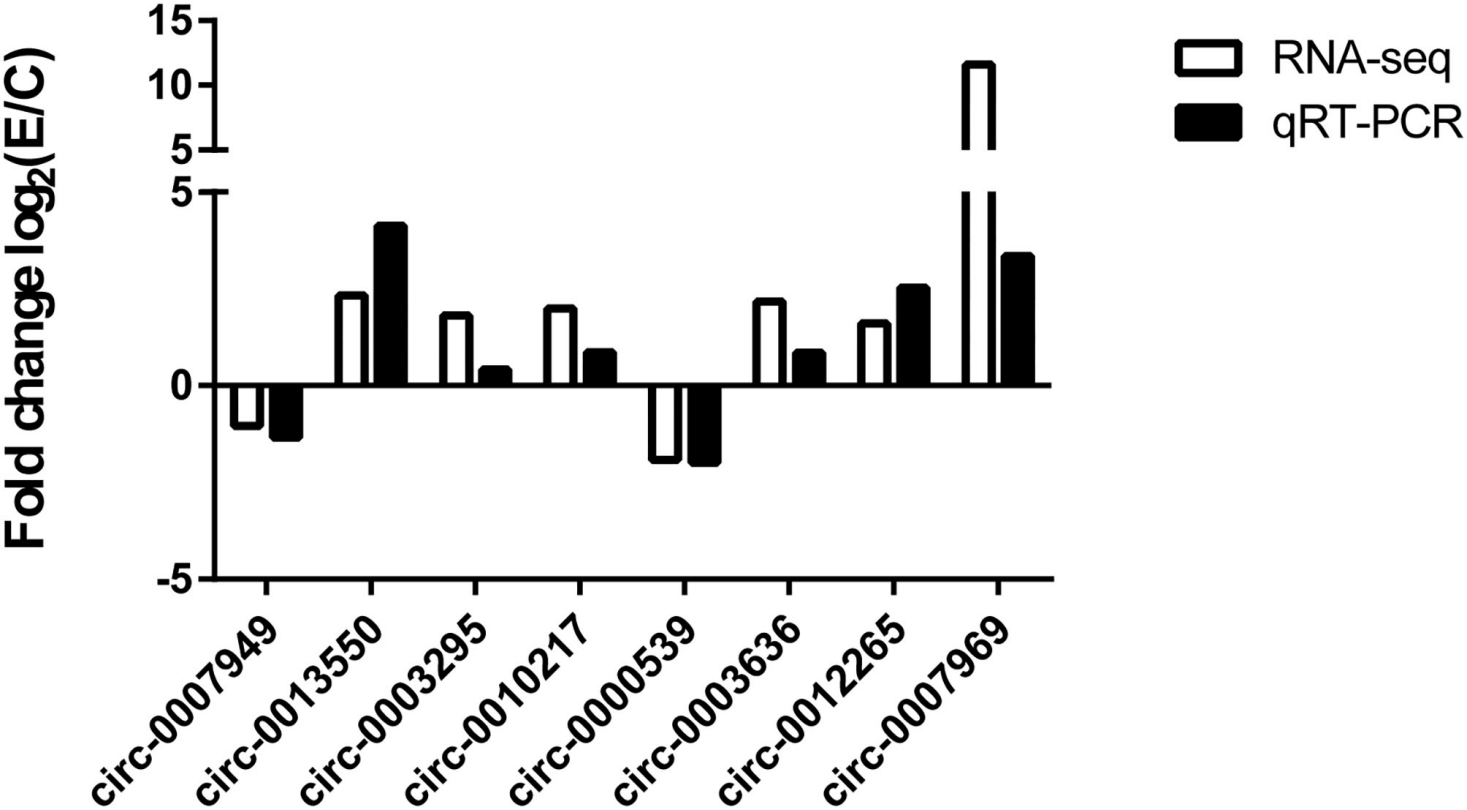
Comparison of results of circRNA sequencing and qRT-PCR. The number of biological replicates for each experiment was 3, and the relative expression levels were normalized to the expression level of GAPDH. Data are presented as means with SD. *P*-values were analyzed by Student's *t*-test.

To validate the circRNA-seq data, some circRNAs (circ-0007949, circ-0013550, circ-0003295, circ-0010217, circ-0000539 circ-0003636, circ-0012265, and circ-0007969) in five groups were randomly selected for qRT-PCR, which belonged to the DECs and circRNA/miRNA interaction network. As shown in Figure 6, the same expression trend of six circRNAs was identified between circRNA-seq data and qRT-PCR data, which revealed that our circRNAs sequencing analysis was highly reliable and accurate (Supplementary Figure S1).

## Discussion

Considering the highly conserved and stable circRNAs, it has become a target for the diagnosis and treatment of many diseases (19–21). A previous study identified that several dysregulated circRNAs were in the murine liver at the early stage of *E. multilocularis* infection, which indicated important roles that DE-circRNAs have played (22). However, the liver is a cellular complex. Multiple cells act different functions in different stages of disease. In this study, DE-circRNAs were identified in multiple cells of the liver, such as HCs, HSCs, and KCs. These findings have been confirmed by qRT-PCR on some selected circRNAs.

Previous studies proved that, during the process of infection, *E. multilocularis* cannot synthesize cholesterol but takes up the lipids derived from the host (23). Furthermore, Xu et al. (24) have identified that mmu-circ-0000081 (circDENND1B) could promote cholesterol efflux by competing the mmu-miR-17-5p with Abca1, while Abca1 participates in cholesterol metabolism. In this study, circDENND1B was significantly upregulated with the log2|FC| of 8.94 at 3 mpi in HCs as the functional cell of liver metabolism, which manifests that *E. multilocularis* infection contributes to the expression of circDENND1B to better absorb cholesterol from host and be conducive to its growth. Undeniably, except for circDENND1B, some other circRNAs were also involved in regulating cholesterol metabolism. mmu-circ-0001679, which had increased expression in HC at 3 mpi, was identified to regulate the expression of nitrogen permease regulator 3-like (NPrl3) (25). Another study proved that NPrl3 may suppress the TOR-dependent transcription of ribosomal protein genes, affect the cell cycle, and result in developmental defects of the internal organs and cardiovascular system of murine (26). Whether *E. multilocularis* infection leads to developmental defects of the liver and cardiovascular system was worth further study. In another study, the downregulated miR-15a-5p of murine liver caused by *E. multilocularis* infection was involved in fatty acid biosynthesis through regulating the expression of acyl-CoA synthetase long-chain family member 1 (ACSL1) and fatty acid synthase (27). In this study, we predict that miR-15a-5p is the target of circ-0001209, which is upregulated at 3 mpi. Whether or not the circ-0001209–miR-15a-5p–ACSL1 axis is involved in the modulation of fatty acid intake from a host infected with *E. multilocularis* needs further study.

Meanwhile, in HSCs, we found that some circRNAs (mmu-circ-0007007, mmu-circ-0013854, mmu-circ-0012341, mmu-circ-00031801, and mmu-circ-0002786) appear with an upregulated expression continually after *E. multilocularis* infection. Although the function of these genes has not been elucidated, some urgent function (promote or suppress hepatic fibrosis) may be played by these circRNAs (12, 28). Additionally, a study in 2021 showed that circPSD3 may suppress the activation and proliferation of HSCs by regulating the expression of miR-92b-3p and Smad7, while in our study, circPSD3 had a downregulated expression in HCs and HSCs, which showed that *E. multilocularis* may alleviate HSC activation and the proliferation of HSCs by reducing the expression of circPSD3 to better parasitize the liver.

In KCs, a total of 429 circRNAs were differentially expressed at 2 mpi—likewise with 331 at 3 mpi. In the network of KCs, the expression of some inflammation-related genes (IL10RA and IL7R) was regulated by circRNAs (circ-0000511, circ-0000149, circ-0000741, and circ-0001684) through sponge miRNAs, which indicated the important role of circRNA in regulating inflammation. Besides this, the circ-0007534–miR-613/SLC25A22 axis is involved in the development of colorectal cancer cells (29), while in KCs, after *E. multilocularis* infection, whether circ-0000511, circ-0000149, circ-0000741, and circ-0001684 regulate the expression of SLC25A22 and are involved in the development of KCs by sponging miR-195a-5p is worth mentioning. Further functional studies would verify these relationships and provide a new perspective to understand the inflammation process caused by *E. multilocularis*.

By analyzing the DECs in three types of cells, we found that only 16 circRNAs were differentially expressed in HCs, HSCs, and KCs, including mmu_circ_0004276, mmu_circ_0000814, mmu_circ_0000058, and so on. Further prediction of the targets of these circRNAs identified that most of these circRNAs had no targets, and only mmu_circ_0000814 and mmu_circ_0000058 had targets. Based on this, we speculated that the DECs played roles mainly in specific cells rather than in all liver cells. The potential regulatory pathways of DECs is still based on HC, HSCs, and KCs.

## Conclusion

Collectively, the present study revealed the circRNA expression profiles of HCs, HSCs, and KCs in murine liver between the infection and control groups and constructed networks among DE-circRNAs, miRNA, and mRNAs, shedding light on the non-coding RNA regulation relationship in different cells of the liver after *E. multilocularis* infection, which provides explanations for the differentially expressed miRNAs in previous studies and the potential way that they may play a role. These data of this study will lay the foundation for further clarification of the pathogenic mechanism of *E. multilocularis* and exploration of new intervention measures.

## Data Availability Statement

The datasets presented in this study can be found in online repositories. The names of the repository/repositories and accession number(s) can be found in the article/Supplementary Material.

## Ethics Statement

The animal study was reviewed and approved by Animal Ethics Committee of Lanzhou Veterinary Research Institute, Chinese Academy of Agricultural Sciences.

## Author Contributions

XL conceived and designed the experiments. TL performed the experiments and drafted the manuscript. TL, LW, HL, YL, GC, GP, and XB analyzed the data. XG, YZ, and XL contributed to reagents and materials. All authors read and approved the final manuscript.

## Funding

This work was funded by the National Key Research and Development Program (No. 2017YFC1601206) and the National Natural Science Foundation of China (No. 32072889).

## Conflict of Interest

The authors declare that the research was conducted in the absence of any commercial or financial relationships that could be construed as a potential conflict of interest.

## Publisher's Note

All claims expressed in this article are solely those of the authors and do not necessarily represent those of their affiliated organizations, or those of the publisher, the editors and the reviewers. Any product that may be evaluated in this article, or claim that may be made by its manufacturer, is not guaranteed or endorsed by the publisher.

## Supplementary Material

Supplementary Figure S1 The relative expression level of 8 circRNAs in 2M-HCs, 2M-HSCs, 3M-HSCs, 2M-KCs, and 3M-KCs were determined by qRT-PCR. The number of biological replicates for each experiment was 3, and the relative expression levels were normalized to the expression level of GAPDH. Data are presented as means with SD. *P*-values were analyzed by Student's *t*-test. ^***^*P* < 0.001, ^**^*P* < 0.01, and ^*^*P* < 0.05.
