# Supplementary figures and images for: circRNA Expression Pattern and circRNA–miRNA–mRNA Network in HCs, HSCs, and KCs of Murine Liver After Echinococcus multilocularis Infection
Source: Front Vet Sci. 2022 Mar 24;9:825307. doi: 10.3389/fvets.2022.825307 (PMC8987445; doi:10.3389/fvets.2022.825307)

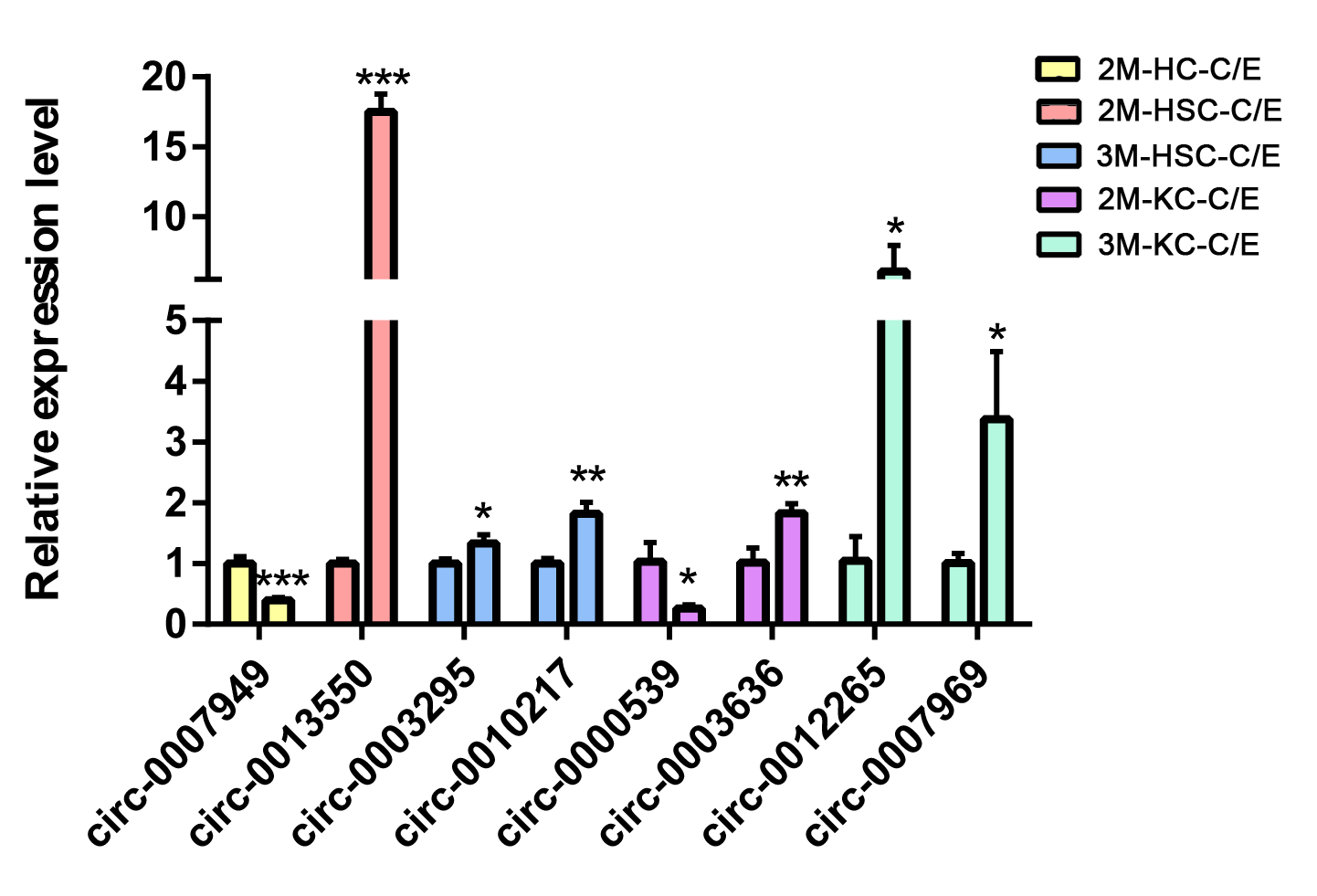

Supplement: Supplementary Figure S1 — The relative expression level of 8 circRNAs in 2M-HCs, 2M-HSCs, 3M-HSCs, 2M-KCs, and 3M-KCs were determined by qRT-PCR. The number of biological replicates for each experiment was 3, and the relative expression levels were normalized to the expression level of GAPDH. Data are presented as means with SD. P-values were analyzed by Student's t-test. ***P < 0.001, **P < 0.01, and *P < 0.05. [file Image_1.tif]
